# ICP-MS-Based Characterization and Quantification of Nano- and Microstructures

**DOI:** 10.3390/nano14070578

**Published:** 2024-03-26

**Authors:** Antonio R. Montoro Bustos

**Affiliations:** The Material Measurement Laboratory, Chemical Sciences Division, National Institute of Standards and Technology (NIST), Gaithersburg, MD 20899, USA; antonio.montorobustos@nist.gov

Since its commercial introduction in the 1980s, inductively coupled plasma mass spectrometry (ICP-MS) has evolved to become arguably the most versatile and powerful technique for the multi-elemental and multi-isotopic analysis of metals, metalloids, and selected non-metals at ultratrace levels. Given its several unique properties, over the last two decades, ICP-MS has become the reference analytical technique for the reliable quantification and elemental composition characterization of nano- and microstructures fostering numerous advanced applications in diverse fields.

This Special Issue includes 12 research and review articles that showcase current developments, fundamental studies, metrological advances, and applications covering a myriad of areas of research associated with the use of ICP-MS for the characterization and quantification of nano- and microstructures, which will give readers a glimpse of the challenges, opportunities, and developmental trends in this field.

The superb capabilities of “stand alone” ICP-MS for the direct ultrasensitive quantification of bulk elemental content, ultratrace elemental impurities, and isotopic determinations have significantly expanded the realm of (bio)analytical applications of nano- and microstructures. Furthermore, ICP-MS has rapidly become an essential on-line element-specific detector for the characterization of polydispersed and complex nano- and microstructures by its hyphenation to most common continuous fractionation/separation techniques. In this context, the potential of ICP-MS, and its combination with different separation techniques, as a complementary tool for the characterization of the nanoparticle (NP) protein corona complexes arising in biological systems is reviewed [1].

The implementation of off-the-shelf ICP-MS instruments, operating at higher time resolution, to perform measurements on individual NPs, commonly called single-particle (SP-)ICP-MS, has swiftly evolved from a technological curiosity to a well-recognized technique for the rapid simultaneous determination of NP size distribution and NP number concentration in very dilute liquid suspensions. A strong indicator of the important role of SP-ICP-MS for the analysis of nano and microstructures is the fact that 10 out of 12 of the articles included in this Special Issue are focused on the advances and applications of SP-ICP-MS.

The remarkable potential of SP-ICP-MS for automation and high-throughput size and isotopic analysis of gold NPs and silver shelled gold core NPs is thoroughly explored through the combination of an innovative autosampler, a high-efficiency sample introduction system, and time-of-flight mass spectrometry (SP-ICP-TOF-MS) [2]. The use of SP-ICP-TOF-MS and SP sector field ICP-MS (SP-SF-ICP-MS) is reported for the challenging analysis of silicon-containing NPs extracted from soil with different extractants in an agricultural application [3]. Alternatively, the high resolution of SP-SF-ICP-MS in combination with conventional sizing techniques enables a deeper understanding of the formation of platinum–palladium NP clusters, offering a rapid and orthogonal characterization of the size and size distribution of this bimetallic NP system [4]. 

Despite the fact that SP-ICP-MS was not considered a priori appropriate for monitoring carbon-based nano- and microstructures, the measurement of microplastics by SP-ICP-MS using carbon isotopic signatures or through metal isotope signatures of doped/functionalized particles is very recently reported in the literature. As an alternative to filtering, a pre-treatment of river water samples with 10% nitric acid for 24 h at room temperature is proposed to improve the detectability of microplastics by SP-ICP-MS. This pre-treatment removed dissolved and particulate carbonate species, oxidating natural organic matter and microorganisms from the river waters, decreasing the high carbon background and improving the accuracy of SP-ICP-MS results [5].

The emerging role of SP-ICP-MS as an invaluable analytical tool for the determination of inorganic NPs in food, food additives, and food relevant matrices is critically reviewed [6]. Over 50 existing studies that employ SP-ICP-MS in this field are overviewed with a particular emphasis on method validation, trends in sample preparation, and spICP-MS methodology. Several key knowledge gaps in this research field are identified, including the direct effects of NPs in food on gastrointestinal tissues and microbiota within the gastrointestinal tract. To address this knowledge gap, the agglomeration behavior and fate of food-grade titanium dioxide (E 171) in human gastrointestinal digestion is rigorously assessed using a thorough multi-technique approach [7]. SP-ICP-MS results reveal that titanium dioxide NPs with a size range that can be adsorbed by the human small intestine can be released into systemic circulation. 

Another relevant knowledge gap with significant commercial, societal, and environment implications is the lack of a full understanding of the short- and long-term impacts of NPs in nano-enabled consumer products. For this reason, the potential of tandem mass spectrometry (SP-ICP-MS/MS) as a fast routine technique for the multi-elemental screening of NPs present in seven powder-based facial cosmetics, purchased from local retailers in the United States, is reported [8]. Surprisingly, the presence of NPs smaller than 100 nm, not disclosed on the ingredient list, is observed in all cosmetic products. Currently the U.S. Food and Drug Administration does not regulate the use of NPs in cosmetics or the disclosure of NPs on packaging. 

Despite considerable progress being made, SP-ICP-MS is still considered an emerging technique, with many advancements yet to come before it can be considered an established methodology. In fact, three examples of metrological advances and efforts to expand the measurement capabilities and consolidate the role of SP-ICP-MS for the characterization of NPs are included in this Special Issue [9,10,11]. The evaluation of SP-ICP-MS size uncertainty of individual NP diameter indicates that the relative uncertainty in the diameter increases as the NP size decreases regardless of the size of the NP calibration standard [9]. Higher precision in the analysis of individual NP diameters can be achieved when using NP calibration standards with a narrower size distribution. A novel approach proposes the use of the bandpass mode for internal standardization in SP-ICP-MS using a platinum NP standard for the characterization of gold NPs in complex matrices [10]. The cited advantages of the internal standard correction in SP-ICP-MS analysis include that the knowledge of the matrix composition is not required, avoiding pre-treatment steps to remove non-spectral interferences, and is less time consuming than standard addition and more universal than isotope dilution analysis. The novel application of isotope dilution analysis in SP-ICP-TOF-MS for the mass and size distribution determination, instead of central tendency, of silver NPs is reported [11]. The use of a TOF mass analyzer enables the simultaneous detection of ^107^Ag^+^ and ^109^Ag^+^ signals in highly diluted suspensions on a particle-by-particle basis, leading to a more precise determination of isotope ratios, which may be relevant for the analysis of complex and/or environmental samples.

Considering the great interest in achieving information from individual cells, the concept behind SP-ICP-MS has been successfully applied to single cells (SC-ICP-MS), enabling the detection and quantification of the metal content within individual cells. In fact, a novel tissue disaggregation protocol is proposed for the first time to expand the capabilities of SC-ICP-MS to analyze single cell suspensions of isolated cells from solid tissues [12].

I would like to personally thank all of the authors and reviewers for their contributions to this Special Issue. The new advances showcased in these studies highlight and consolidate the pivotal role of ICP-based technologies in the development of many real-world applications of nano- and microstructures that address new scientific and societal challenges. Hopefully, the research outlined in this Special Issue can inspire forthcoming investigations toward the advancement of new paths to strengthen the role of ICP-MS in this field.

## References

[1] Fuentes-Cervantes A., Ruiz Allica J., Calderón Celis F., Costa-Fernández J.M., Ruiz Encinar J. (2023). The Potential of ICP-MS as a Complementary Tool in Nanoparticle–Protein Corona Analysis. Nanomaterials.

[2] Manard B.T., Bradley V.C., Quarles C.D., Hendriks L., Dunlap D.R., Hexel C.R., Sullivan P., Andrews H.B. (2023). Towards Automated and High-Throughput Quantitative Sizing and Isotopic Analysis of Nanoparticles via Single Particle-ICP-TOF-MS. Nanomaterials.

[3] Li Z., Hadioui M., Wilkinson K.J. (2023). Extraction of Silicon-Containing Nanoparticles from an Agricultural Soil for Analysis by Single Particle Sector Field and Time-of-Flight Inductively Coupled Plasma Mass Spectrometry. Nanomaterials.

[4] Martinez-Mora O., Tirez K., Beutels F., Brusten W., Leon-Fernandez L.F., Fransaer J., Dominguez-Benetton X., Velimirovic M. (2023). Exploring Pt-Pd Alloy Nanoparticle Cluster Formation through Conventional Sizing Techniques and Single-Particle Inductively Coupled Plasma—Sector Field Mass Spectrometry. Nanomaterials.

[5] Trujillo C., Pérez-Arantegui J., Lobinski R., Laborda F. (2023). Improving the Detectability of Microplastics in River Waters by Single Particle Inductively Coupled Plasma Mass Spectrometry. Nanomaterials.

[6] Loeschner K., Johnson M.E., Bustos A.R.M. (2023). Application of Single Particle ICP-MS for the Determination of Inorganic Nanoparticles in Food Additives and Food: A Short Review. Nanomaterials.

[7] Ferraris F., Raggi A., Ponti J., Mehn D., Gilliland D., Savini S., Iacoponi F., Aureli F., Calzolai L., Cubadda F. (2023). Agglomeration Behavior and Fate of Food-Grade Titanium Dioxide in Human Gastrointestinal Digestion and in the Lysosomal Environment. Nanomaterials.

[8] Hebert D., Nelson J., Diehl B.N., Zito P. (2023). Single-Particle ICP-MS/MS Application for Routine Screening of Nanoparticles Present in Powder-Based Facial Cosmetics. Nanomaterials.

[9] Yamashita S., Miyashita S.-I., Hirata T. (2023). Size Uncertainty in Individual Nanoparticles Measured by Single Particle Inductively Coupled Plasma Mass Spectrometry. Nanomaterials.

[10] Aramendía M., Leite D., Resano J., Resano M., Billimoria K., Goenaga-Infante H. (2023). Isotope Dilution Analysis for Particle Mass Determination Using Single-Particle Inductively Coupled Plasma Time-of-Flight Mass Spectrometry: Application to Size Determination of Silver Nanoparticles. Nanomaterials.

[11] Bazo A., Aramendía M., Nakadi F.V., Resano M. (2023). An Approach Based on an Increased Bandpass for Enabling the Use of Internal Standards in Single Particle ICP-MS: Application to AuNPs Characterization. Nanomaterials.

[12] Álvarez-Fernández García R., Gutiérrez Romero L., Bettmer J., Montes-Bayón M. (2022). Capabilities of Single Cell ICP-MS for the Analysis of Cell Suspensions from Solid Tissues. Nanomaterials.

